# Rapid Mining of Candidate Genes for Verticillium Wilt Resistance in Cotton Based on BSA-Seq Analysis

**DOI:** 10.3389/fpls.2021.703011

**Published:** 2021-10-08

**Authors:** Yanli Cui, Qun Ge, Pei Zhao, Wei Chen, Xiaohui Sang, Yunlei Zhao, Quanjia Chen, Hongmei Wang

**Affiliations:** ^1^Engineering Research Centre of Cotton, Ministry of Education, Xinjiang Agricultural University, Ürümqi, China; ^2^State Key Laboratory of Cotton Biology, Institute of Cotton Research, Chinese Academy of Agricultural Sciences, Anyang, China

**Keywords:** upland cotton, BSA-seq, verticillium wilt, QTL, resistance gene

## Abstract

Cotton is a globally important cash crop. Verticillium wilt (VW) is commonly known as “cancer” of cotton and causes serious loss of yield and fiber quality in cotton production around the world. Here, we performed a BSA-seq analysis using an F_2:3_ segregation population to identify the candidate loci involved in VW resistance. Two QTLs (qvw-D05-1 and qvw-D05-2) related to VW resistance in cotton were identified using two resistant/susceptible bulks from the F_2_ segregation population constructed by crossing the resistant cultivar ZZM2 with the susceptible cultivar J11. A total of 30stop-lost SNPs and 42 stop-gained SNPs, which included 17 genes, were screened in the qvw-D05-2 region by SnpEff analysis. Further analysis of the transcriptome data and qRT-PCR revealed that the expression level of *Ghir_D05G037630* (designated as *GhDRP*) varied significantly at certain time points after infection with *V. dahliae*. The virus-induced gene silencing of *GhDRP* resulted in higher susceptibility of the plants to *V. dahliae* than the control, suggesting that *GhDRP* is involved in the resistance to *V. dahlia* infection. This study provides a method for rapid mining of quantitative trait loci and screening of candidate genes, as well as enriches the genomic information and gene resources for the molecular breeding of disease resistance in cotton.

## Introduction

As a globally important cash crop, cotton provides approximately 35% of total fiber used worldwide (Zhang et al., 2014a) and is also an important source of oilseed (Hulse-Kemp et al., 2015). Verticillium wilt (VW) is commonly known as a “cancer” of cotton, and it is one of the most destructive diseases caused by the soil-borne fungus *Verticillium dahliae Kleb* (Burpee and Bloom, 1978; Fradin et al., 2011). VW not only reduces the yield of cotton but also causes significant degradation of the fiber quality (Zhang et al., 2014a). Development of new cotton varieties resistant to VW has been considered as the most effective and feasible way to control VW (Zhao et al., 2017).

It has been widely recognized that VW is a quantitative trait controlled by multiple genes (Devey and Roose, 1987; Wang et al., 2004, 2010). The quantitative trait loci (QTL) related to VW in cotton have been located using F_2_ or recombinant inbred line (RIL) populations. For example, Fang et al. (2001) found a RAPD marker with OPB-19_1300_ that is related to wilt resistance in upland cotton by bulked segregant analysis (BSA). Zhen et al. (2006) screened 768 pairs of SSR primers with BSA by using the upland cotton cultivar CCRI8 and the island cotton cultivar Pima90-53 as parents, and identified the genetic distance between the locus of VW resistance and BNL3255-208 marker as 13.7cM. Shi et al. (2016) used the BC_1_F_1_, BC_1_S_1_, and BC_2_F_1_ populations generated from the cross between Hai1 (an island cotton cultivar with high VW resistance) and CCRI36 (a susceptible upland cotton cultivar) to locate the QTLs related to VW resistance of cotton. Further, they constructed a linkage map containing 2,292 SSR marker sites with a total length of 5115.16cM, and detected 48 QTLs related to VW resistance. Localization of these QTLs lays a solid research basis for the fine mapping and cloning of disease resistant genes. To date, many genes related to VW resistance have been cloned in cotton, such as *GbRVd* (Yang et al., 2016), *GbaNA1* (Li et al., 2018), *GbSTK* (Zhang et al., 2013), *GbRLK* (Jun et al., 2015), and *Gbvdr3* (Chen et al., 2016), which were derived from island cotton, and *GhBAK1* (Gao et al., 2013), *GhPAO* (Mo et al., 2015), *GhSKIP35* (Zhang et al., 2017), and *GhPGIP1* (Liu et al., 2017), which were derived from upland cotton. Most of these genes encode enzymes and receptor proteins with certain roles in disease resistance. Most research on VW resistance genes has been performed using reverse genetics; however, only a few genes have been cloned through forward genetics.

BSA, a method of forward genetics, is a practical technique of gene/marker mapping to identify the genomic regions that contain the loci affecting a certain trait. As early as 1991, Michelmore et al. (1991) used BSA to successfully screen three markers closely associated with the downy mildew resistance gene DM5/8 in an isolated lettuce population. Combined with deep sequencing, BSA-seq analysis can realize efficient and accurate fine mapping of QTLs or key genes related to some important traits of crops. Currently, BSA-seq analysis is widely used in the research on various agronomic traits of different crops such as maize (Klein et al., 2018), apple (Jia et al., 2018), chickpea (Singh et al., 2016), pepper (Lee et al., 2020), and cotton (Zhu et al., 2017), and the candidate gene for pepper fruit color (*CaPRR2*) and the bud yellow gene (*GhCHL1*) of cotton have been successfully identified.

In the research on plant disease resistance, QTLs for related traits have been identified and even the candidate genes have been screened by BSA-seq analysis, such as peanut rust and late spot (Pandey et al., 2017; Clevenger et al., 2018), tomato yellow rot (Zhao et al., 2016), corn gray leaf spot (Cui et al., 2018), and crustal blight of chickpea (Deokar et al., 2019). However, BSA-seq analysis has not been used in the detection of candidate genes for VW resistance in cotton, possibly due to the complexity of the cotton genome.

With the availability of the draft cotton genome (Wang et al., 2018), map-based gene cloning technology has been greatly developed in cotton. On this basis, we combined BSA-seq analysis and virus-induced gene silencing (VIGS) for the rapid mapping and identification of candidate genes associated with VW resistance in cotton. This work provides a paradigm for rapid map-based gene cloning in plants with large and polyploid genomes.

## Materials and Methods

### Plant Materials and Trait Evaluation

The cotton F_2_ segregation population was developed using the VW resistant cultivar Zhongzhimian2 (ZZM2) and susceptible cultivar Jimian11 (J11) as cross parents in 2014. To evaluate the VW resistance, 232 F_2:3_ cotton lines together with their parents were planted in a field in Xinjiang (41.29°N, 80.24°E, a serious verticillium disease plot), where the phenotypes were observed from July to September at the adult-plant stage in 2017, and in a greenhouse in Henan Anyang (36.1°N, 114.35°E), where the phenotypes were observed at the seedling stage in 2018. In the greenhouse experiments, phenotypes were observed at 15–35days post inoculation (dpi) with the *V. dahliae* isolate Vd080, a defoliating strain with moderate pathogenicity to cotton in the two-leaf stage. The greenhouse had a controlled 12h photoperiod and temperature variation of 23–30°C. The experiments in both environments were of a randomized block design with three replications. VW resistance was evaluated with the disease index (DI), which could present a comprehensive and objective measurement of plant health and was classified into five grades according to the symptoms on the cotyledons and true leaves (Jian et al., 2001; Zhu et al., 2010; Zhang et al., 2012).

The susceptible cultivar J11 was used as a susceptible control to estimate the severity of disease and determine the optimal time for investigation. The DI was further adjusted into the relative disease index (RDI) according to previous descriptions (Zhao et al., 2014). A higher DI or RDI value indicates a more advanced infection by *V. dahliae*. Descriptive statistics, analysis of variance (ANOVA), and correlation analysis were performed to evaluate the traits performance in the greenhouse and field using the SAS system (v8.02). Origin8.0 software was used to present the phenotypic differences between the two extreme pools.

### BSA-Seq Library Construction and Analysis

To detect variations in the genome related to VW resistance, a BSA-seq analysis was implemented using two extreme pools. Eighteen resistant F_2_ individuals (R-bulk) and 18 susceptible F_2_ individuals (S-bulk) in both environments were selected for pooling and sequencing. DNA from the two pools was extracted with the SDS method as previously described (Kuang et al., 2010), quantified using a Nanodrop 2000 spectrophotometer (Thermo Fisher, United States), detected by 1% agarose gel electrophoresis, and then mixed at equal concentrations, respectively. The DNA of the two extreme pools and their parents was used to prepare four DNA libraries for Illumina sequencing on the Illumina HiSeq2500 platform at BGI (Beijing Genomics Institute, Beijing, China). The quality of the raw data obtained from sequencing was evaluated and clean reads were obtained through filtration. Clean data were compared to the reference genome (Wang et al., 2018) using the BWA software (Md et al., 2019). Based on the results, SNPs and InDels were detected and annotated using the GATK4.0 software.^1^ The QTL interval was obtained according to the △SNP-index and G-value, which were calculated using the QTLseqr software (Mansfeld and Grumet, 2018).

### SnpEff Analysis

Stop-gained and stop-lost SNPs, respectively, with generation of new stop codons and loss of stop codons may have significant effects on gene function. Therefore, in this study, stop-gained and stop-lost SNPs in the QTL interval were screened using the SnpEff software (Klein et al., 2018; https://pcingola.github.io/SnpEff/).

### Structural Analysis of Candidate Genes

In order to analyze the structure of the candidate gene *Ghir_D05G037630* and its difference between ZZM2 and J11, the DNA was divided into three segments for amplification because of the long length of the gene. Primer Premier 5.0 software was used for primer design and the primer sequences are shown in Supplementary Table 1. The DNA was amplified using high fidelity enzyme KOD-Plus-Neo (KOD-401, Toyobo, Japan) and its PCR products were purified by 1% concentration agarose gel electrophoresis and recycled using a Vazyme’s FastPure Gel DNA Extraction Mini Kit (DC301, Vazyme, Nanjing, China). The target fragment was connected and recombined with the carrier using a 5-min TA/Blunt-Zero Cloning Kit (C601, Vazyme). The recombinant product was transformed into competent cells DH5a, and monoclones grown from expanded culture were sent to Sangon (Sangon Biotech, Zhengzhou Branch, China) for sequencing. The SeqMan software (v7.1.0) was used to splice and align the sequences.

### Preparation of Samples Infected by *V. dahliae*

The cultivars ZZM2 and J11 were planted in an incubator under a 16h/light/25°C and 8h/dark/22°C regimen and infected with Vd080 at the three-leaf stage as previously described (Zhu et al., 2010). Root tissue samples were taken at 0, 6, 12, 24, 36, 48, 72, and 96h after infection and then stored at −80°C. Total RNA was extracted using an RNA prep Pure Plant Kit (DP441, Tiangen, Beijing, China) and used to generate cDNA with a PrimeScript RT reagent kit (RR037A, Takara, Japan) following the manufacturer’s instructions.

### Quantitative Real-Time PCR

The *Gossypium hirsutum histone H3.3* gene (*Ghir_D03G004040*) was used as an internal control for cotton. Diluted cDNA was used for qRT-PCR with ChamQ Universal SYBR qPCR Master Mix (Q711, Vazyme) on a QuantStudio 6 Flex fluorescence quantitative PCR instrument (Applied Biosystems, USA). A two-step method was used with the following PCR conditions: 95°C for 30s, 40cycles of 95°C for 10s, and 60°C for 30s. The dissociation curves of each reaction were checked, and the cycle threshold (CT) 2^−△△CT^ method (Livak and Schmittgen, 2001) was used to calculate the expression level of each target gene. Each reaction was performed with at least three biological replicates.

### VIGS Vector Construction and Pathogen Inoculation

The VIGS experiments were carried out with the method described by Pang et al. (2013). The primer pair Gh_V037630-2F/R was designed at 1931–2280bp of *GhDRP* (Supplementary Table 1) and used to amplify the specific fragment by polymerase chain reaction (PCR) from ZZM2. The target fragment was linked to the *Tobacco Rattle Virus* (TRV)-based vector (Bachan and Dinesh-Kumar, 2012; Zeng et al., 2019) at the BamHI and SacI restriction sites by homologous recombination using a ClonExpress II One Step Cloning Kit (C112, Vazyme). All vectors used in this study were transformed into *Agrobacterium tumefaciens* strain GV3101 using the freeze–thaw method (Gong et al., 2018). The cotyledons of 11-day-old seedlings of ZZM2 were injected with equal amounts of TRV vectors. After 24h of incubation in darkness, the cotton seedlings were transferred to the greenhouse (Gong et al., 2017). Approximately 10days after VIGS injection, when the true leaves in TRV-PDS (positive control) began to fade and turn white, leaves from the TRV-target and TRV-empty seedlings were collected for each of the three replicates. Total RNA was extracted and reversed into cDNA for qRT-PCR to detect the silencing efficiency using the primer pair Ghir_RT037630F/R in Supplementary Table 1. After the silencing of the target gene was confirmed, the cotton seedlings were inoculated with *V. dahliae*.

### Quantification of *V. dahliae* Colonization and Recovery Assay

qRT-PCR approach was performed to detect and quantify *V. dahliae* colonization. Leaves from each of three independent plants of TRV::00 and TRV::*GhDRP* were sampled at 20 and 25 dpi, rapidly frozen in liquid nitrogen, and stored at −80°C. The primers VdActin-F/R (Supplementary Table 1) and the qRT-PCR program were used as described by Atallah et al. (2007). To visualize the degree of *V. dahliae* infection, a recovery assay was performed using fragments from the first node of the stem (Zhang et al., 2011). The stem segment surface was sterilized with 70% ethanol for 20–30s, sodium hypochlorite for 1min, and sterile water for 4–5 times, and then placed on potato dextrose agar (PDA) culture medium, which were incubated at 25°C under dark conditions for 2–3 d. The sensitivity of cotton to *V. dahliae* was evaluated according to the degree of fungal growth in stem segments.

### Cell Death Assay

Cell death in cotton leaves at 25 dpi was visualized by trypan blue staining. Leaves were soaked in trypan blue dye (10ml phenol, 10ml glycerol, 10ml lactic acid, 10ml water, and 0.02g of trypan blue; then dilution with 96% ethanol in a ratio of 1:2), boiled for 1min, cooled to room temperature, and soaked overnight. The next day, the samples were decolorized with a chloral hydrate solution (2.5g/ml) for phenotype observation and photo taking.

### Safranin-Fixed Green Dyeing Assay

Safranin-fixed green dyeing could highlight the tissues that have undergone lignification or suberification, along with the cellulose cell walls of vascular plants. An upper stem segment of approximately 4cm was taken at 25 dpi and the cross section was stained with safranin-fixed green dye to observe cell staining and determine the sensitivity of cotton to *V. dahliae*. The experiment was performed by Servicebio (Wuhan, China).

## Results

### Phenotyping of Verticillium Wilt Resistance in F_2:3_ Population

**Figure 1:**
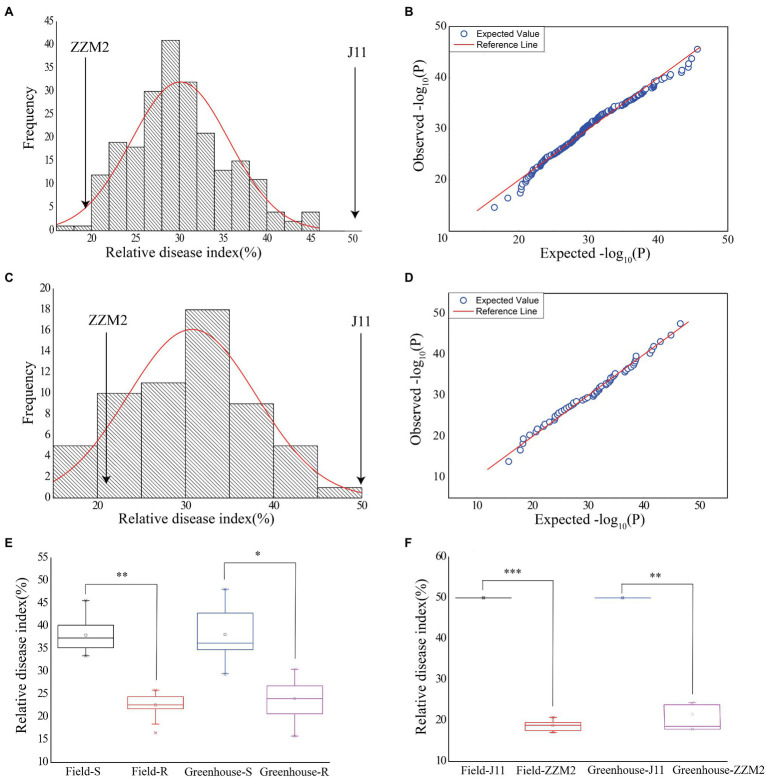
Phenotypic variations of RDI in the field and greenhouse. **(A)** Normal distribution diagram of the RDI in the field. **(B)** Quantile-quantile plot for RDI in the field. **(C)** Normal distribution diagram of the RDI in the greenhouse. **(D)** Quantile-quantile plot for RDI in the greenhouse. **(E)** Box plot for the RDI of selected individuals in two pools in the two environments. Greenhouse-R stands for the RDI of 18 resistant individuals in the greenhouse. Field-R stands for the RDI of 18 resistant individuals in the field. Greenhouse-S stands for the RDI of 18 susceptible individuals in the greenhouse. Field-S stands for the RDI of 18 susceptible individuals in the field. **(F)** Box plot for the RDI of the two parents in the two environments. Asterisk indicates statistically significant differences determined by Student’s t-test (*p < 0.05; **p < 0.01; ***p < 0.001).

Assessment of VW severity showed that the RDI of the parent ZZM2 was 18.71 in the field and 21.25 in the greenhouse, respectively; and that of J11 was adjusted to 50 in both environments, which was used as a susceptible control (Figure 1F). There were considerable variations in RDI in the F_2:3_ population, ranging from 16.31 to 45.31 (30.81 in average) in the field, and from 15.79 to 46.56 (31.17 in average) in the greenhouse (Table 1). The ANOVA results revealed that different genotypes had significantly different RDI whether in the field (*F* value=3.24, *p*<0.0001) or greenhouse (*F* value=11.35, *p*<0.0001; Table 1). The values of RDI in the two environments followed a normal distribution (Figures 1A,C). A quantile-quantile (QQ) plot showed that the observed *p* values were consistent with the expected *p* values in the field (Figure 1B) and greenhouse (Figure 1D). Correlation analysis showed that the RDI value in the greenhouse was strongly correlated with that in the field (*r*=0.628, *p*<0.001), which revealed that RDI investigated in seedling and adult stages could reflect the resistance of cotton to VW.

**Table 1:** Descriptive statistics and ANOVA results of VW resistance (indicated by RDI) in the field and greenhouse environments.

Environment	Mean	SD	Variance	Min	Max	CV (%)	Skewness	Kurtosis	DF	Anova SS	Mean Square	F Value	Pr>F
field	30.81	5.58	31.15	16.31	45.31	18.54	0.40	−0.05	223	11374.34	51.00	3.24	<0.0001
greenhouse	31.17	7.30	53.34	15.79	46.56	23.75	−0.01	−0.63	58	7005.58	120.79	11.35	<0.0001

Eighteen F_2_ plants with the lowest and highest RDI, respectively, were selected to construct two extreme pools, in which the individuals had stable performance regardless of the two assessment method in the field and greenhouse, and the RDI of ZZM2 and the R-bulk was significantly lower than that of J11 and the S-bulk (Figure 1E; Supplementary Table 2). These results indicated that the experimental materials met the requirements of BSA-seq analysis.

### Selection of Candidate QTLs

**Figure 2:**
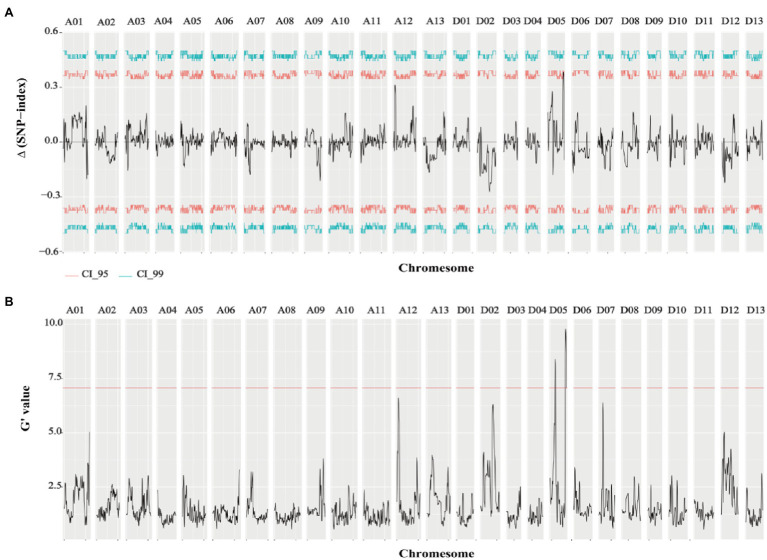
Results of △SNP-index and G-value of VW resistance extreme pools. **(A)** Results of △SNP-index in VW resistant extreme pools. **(B)** Results of G-value in VW resistant extreme pools (*q*=0.01).

Genome-wide sequencing was carried out in the two extreme pools with a 20x depth, as well as for the two parents. Clean data (294.19G) were obtained from the raw data (306.41G) by filtering, including 80.62G reads for ZZM2, 69.54G reads for J11, 72.36G reads for R-bulk, and 71.67G reads for S-bulk (Supplementary Table 3). After the sequencing, 99.42–99.81% of sequences could be successfully mapped to the reference genome (Wang et al., 2018; Supplementary Table 4). Therefore, this protocol could be used for subsequent variation detection and gene mapping of traits. QTLseqr is an R package used to identify QTLs by combining QTL-seq and G’ approach. Candidate QTL segments were determined by using a q-value ≥0.01 in G’ or an △SNP-index ≥99% in the QTL-seq. As a result, two QTLs (18.67–20.74Mb, 2.06Mb in length; 58.19–62.88Mb, 4.69Mb in length) located on chromosome D05 related to VW resistance were obtained (Figure 2; Table 2).

**Table 2:** Information of the candidate QTLs of verticillium wilt resistance.

CHROM	qtl	start	end	length	nSNPs	avgSNPs_Mb	meanPval	meanQval
Ghir_D05	qvw-D05-1	18,676,998	20,741,557	2,064,559	1,001	485	2.74E-05	0.002605994
Ghir_D05	qvw-D05-2	58,195,994	62,887,284	4,691,290	7,777	1,658	1.30E-05	0.001653478

### SnpEff Analysis

A total of 123 stop-gained SNPs and 99 stop-lost SNPs were found through the annotation of 69,642 SNPs on chromosome D05 using the SnpEff software. There were 42 stop-gained SNPs and 30stop-lost SNPs in the region of qvw-D05-2, while none located within the qvw-D05-1 interval. These 72 mutation sites, which involve 17 genes in total, were distributed between 60,016,518bp and 61,342,887bp (Table 3).

**Table 3:** Seventeen genes involving 72 mutation sites in the qvw-D05-2 interval.

Gene ID	Stop-lost SNPs	Stop-gained SNPs
Ghir_D05G037600		60,016,518
Ghir_D05G037610	60,028,687,60,029,472,60,029,916, 60,029,917,60,029,993,60,030,399, 60,030,407,60,031,346,60,031,431, 60,031,591,60,031,644,60,031,655, 60,031,718,60,031,855	60,029,070,60,029,308,60,029,311, 60,029,352,60,029,362,60,029,845, 60,029,983,60,030,137,60,030,170, 60,030,389,60,030,484,60,030,599, 60,031,032,60,031,295,60,031,762, 60,031,928,
Ghir_D05G037620	60,038,613	60,038,430
Ghir_D05G037630		60,063,746,60,069,431
Ghir_D05G037640		60,098,573,60,098,639
Ghir_D05G037650	60,116,797	60,118,071
Ghir_D05G037670	60,277,261,60,280,293,60,280,721, 60,280,827	60,283,114,60,283,152
Ghir_D05G037690	60,304,322	60,308,519,60,308,605
Ghir_D05G037740	60,392,358	60,392,305,60,392,611
Ghir_D05G037820	60,471,685	
Ghir_D05G037850	60,574,375,60,575,359,60,575,360, 60,575,362,60,575,429	60,572,956,60,574,925,60,575,169, 60,575,298,60,576,319,60,576,329
Ghir_D05G037880		60,686,560
Ghir_D05G038110		61,099,420,61,099,920,61,100,203
Ghir_D05G038190		61,173,648
Ghir_D05G038210	61,196,009	61,196,394
Ghir_D05G038220		61,200,375
Ghir_D05G038360	61,342,887	

### Screening of Candidate Genes

**Figure 3:**
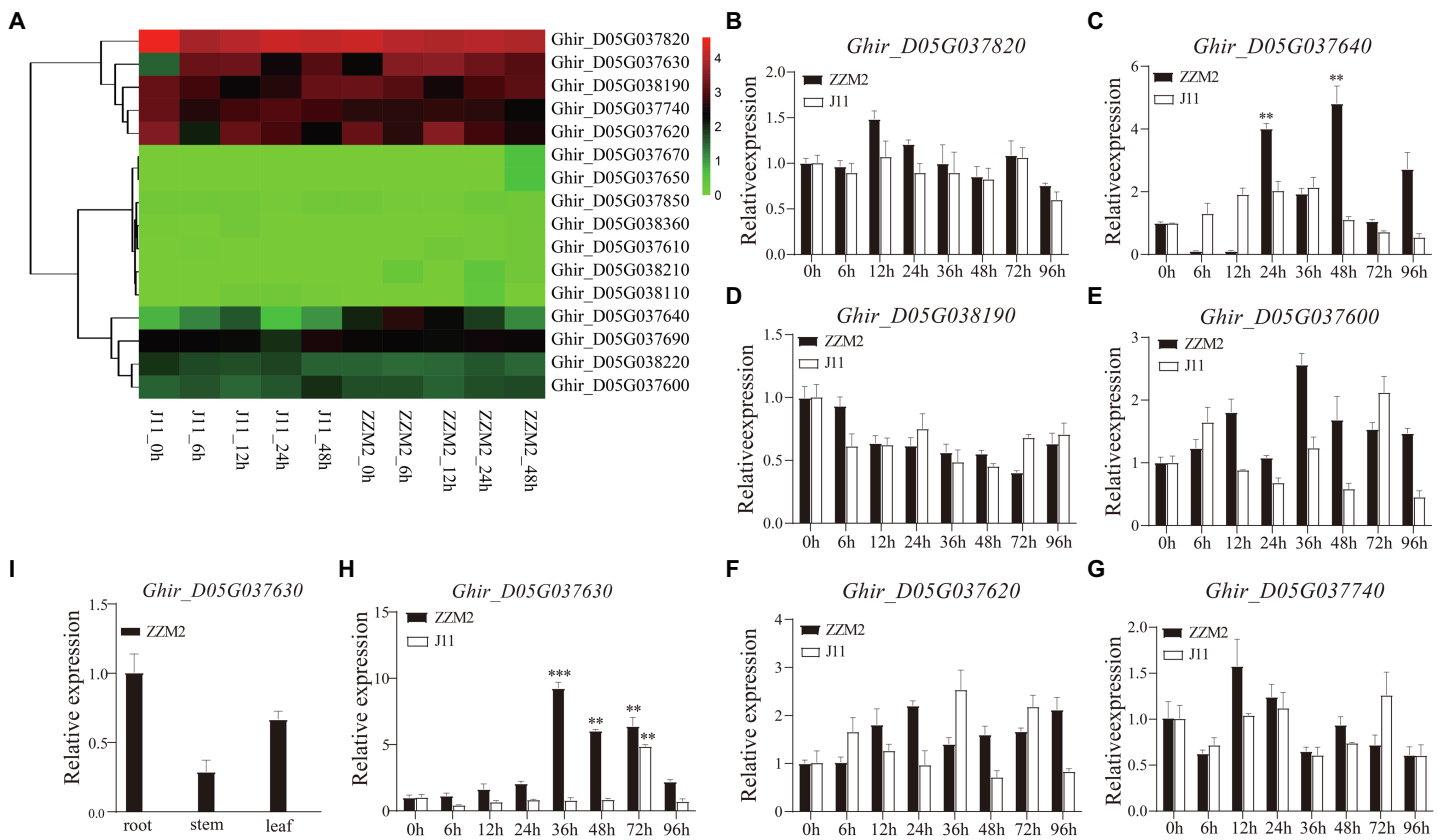
Differential expression of candidate genes. **(A)** Heatmap of the 17 candidate genes in transcriptome analysis. **(B–H)** Expression patterns of the seven candidate genes at 0, 6, 12, 24, 36, 48, 72, and 96h after infection with *V. dahliae* in root of ZZM2 and J11. **(I)** Expression patterns of *Ghir_D05G037630* in root, stem, and leaf in ZZM2. The error bar represents the standard deviation of three biological replicates. Asterisk indicates statistically significant differences determined by Student’s t-test (^**^p<0.01; ^***^p<0.001).

Combined with the transcriptome data from our previous study (Zhao et al., 2021), seven genes were found to be significantly differentially expressed, including *Ghir_D05G037820*, *Ghir_D05G037630*, *Ghir_D05G038190*, *Ghir_D05G037740*, *Ghir_D05G037620*, *Ghir_D05G037640*, and *Ghir_D05G037600* (Figure 3A). To determine whether these seven genes were related to VW resistance, we performed a qRT-PCR analysis to detect candidate genes expression at 0, 6, 12, 24, 36, 48, 72, and 96h after infection with *V. dahliae* in root of ZZM2 and J11. The qRT-PCR results showed that two genes (*Ghir_D05G037630* and *Ghir_D05G037640*) were up-regulated significantly after *V. dahliae* inoculation in ZZM2, of which *Ghir_D05G037630* showed the most significant differential expression between the two parents at different stages (Figures 3B–H). Tissue pattern analysis showed that the expression level of *Ghir_D05G037630* in root was higher than that in leaf and stem in ZZM2 (Figure 3I). *Ghir_D05G037630* is homologous to *AT4G27220* in *Arabidopsis*, which is annotated as a disease-resistant protein. Therefore, we mainly focused on the candidate gene *Ghir_D05G037630* and designated it as *GhDRP*.

The DNA of *GhDRP* had a total length of 7,558bp including six exons and five introns, while the CDS sequence had a total length of 3,876bp, and the protein sequence contained 1,291 amino acids, including one NB-ARC and seven LRR domains. *GhDRP* was cloned from ZZM2 and J11, respectively, and the results showed that there was a mutation of one base, namely base “C” in ZZM2 and base “A” in J11 at 301 bp of the gene, which caused a change in amino acids to produce proline in ZZM2 and glutamine in J11 (Supplementary Figure 1). The mutated amino acid was located on the Pfam domain PF00931 (NB-ARC domain), which is related to plant disease resistance. These results indicated that *GhDRP* may play an important role in VW resistance of cotton.

### Effect of *GhDRP* Silencing on VW Resistance of Cotton

**Figure 4:**
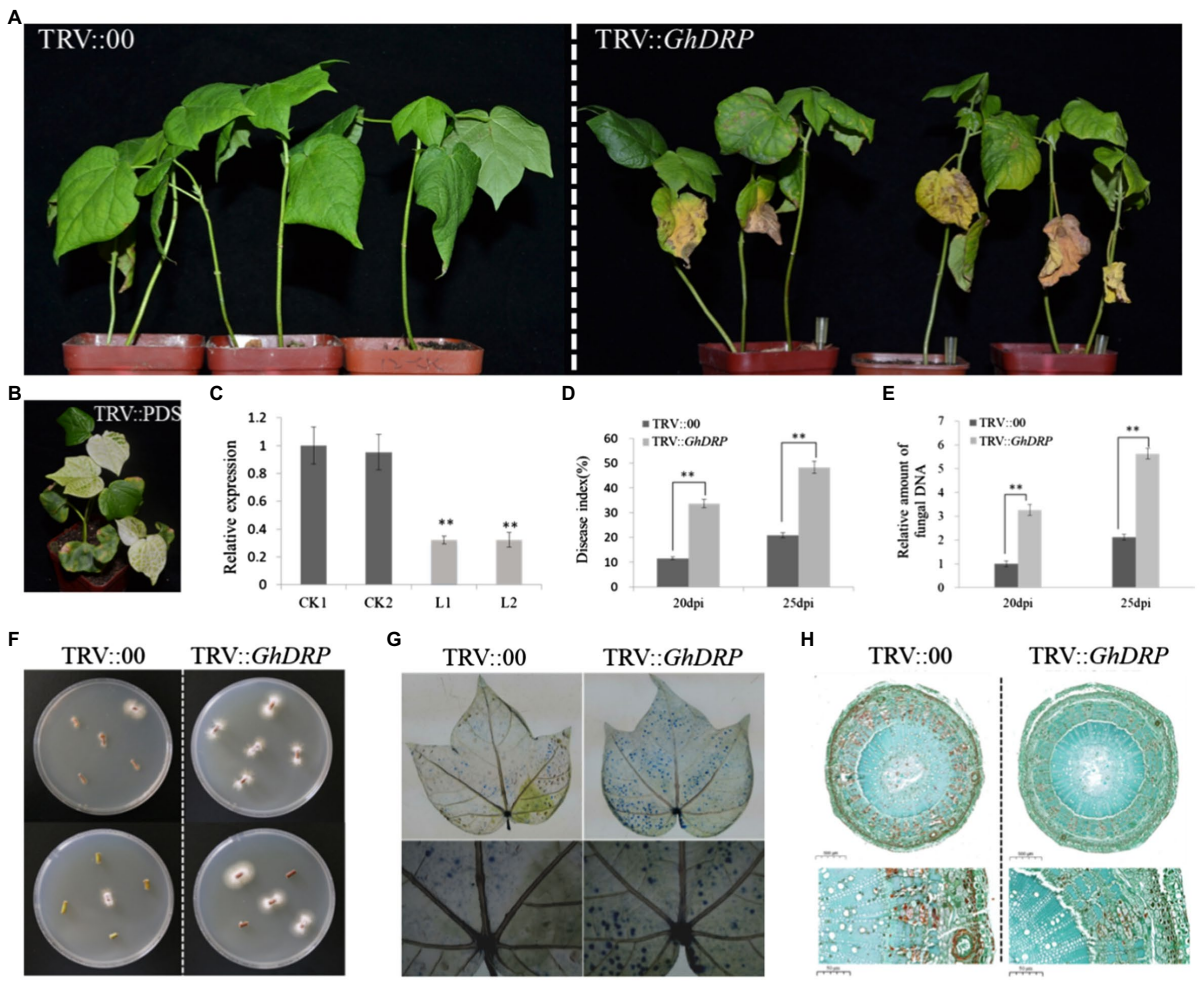
Effect of *GhDRP* silencing on *V. dahliae* resistance of cotton. **(A)** Phenotypes of TRV::*GhDRP* and TRV::00 plants induced by *V. dahliae* at 25 dpi. **(B)** Phenotype of positive control. **(C)** Transcript levels of *GhDRP* in the leaves of TRV::*GhDRP* and TRV::00 plants. CK1 and CK2 represent the control plants TRV::00; L1 and L2 represent the gene silencing plants TRV::*GhDRP*. Asterisk indicates statistically significant differences determined by Student’s *t*-test (^**^p<0.01), and the same below. **(D)** DI in TRV::*GhDRP* and TRV::00 plants at 20 dpi and 25 dpi. **(E)** Relative amount of fungal DNA in the leaves of TRV::*GhDRP* and TRV::00 plants at 20 dpi and 25 dpi. **(F)** The fungi in the stem segment were restored on PDA culture medium. **(G)** Trypan blue staining of the leaves of TRV::*GhDRP* and TRV::00 plants at 25 dpi. **(H)** Cell morphology of the stem cross section of TRV::*GhDRP* and TRV::00 plants at 25 dpi.

In order to further investigate the function of *GhDRP* in the VW resistance of cotton, the VIGS system was used to generate *GhDRP*-knockdown plants. Approximately 10days after the injection of the VIGS vector, the newly grown true leaves of the PDS control plants faded and gradually turned white (Figure 4B), indicating successful injection of the VIGS vector. Leaves of TRV::*GhDRP* plants and TRV::00 plants were randomly selected to measure the expression level of *GhDRP* by qRT-PCR. The results showed that the expression level of *GhDRP* in TRV::*GhDRP* plants was significantly lower than that in the control (Figure 4C), indicating the successful silencing of *GhDRP*. After inoculation with *V. dahliae* for 25days, yellowing and then peeling of leaves were observed in TRV::*GhDRP* plants, while no apparent symptoms of disease were observed in TRV::00 plants (Figure 4A).

The DI value of TRV::*GhDRP* plants was significantly higher than that of the control at 20 and 25 dpi (Figure 4D). The qRT-PCR results showed that *V. dahliae* biomass in TRV::*GhDRP* plants was significantly higher than that in the control (Figure 4E), indicating that the plants with the silencing of *GhDRP* were more susceptible to *V. dahliae*. The recovery assay revealed that significantly more fungal colonies were present in TRV::*GhDRP* plants than in the control plants (Figure 4F). Trypan blue staining was then performed to compare the leaves of plants under different treatments at 25 dpi. The leaves from TRV::*GhDRP* plants exhibited large areas of staining and significant cell damage or death, while those from the control plants showed smaller areas of staining and less severe cell damage (Figure 4G). Examination of the cell morphology after pathogen inoculation showed that the cells of TRV::00 plants underwent more severe lignification than those of TRV::*GhDRP* plants (Figure 4H), suggesting that the plants might resist *V. dahliae* infection through lignification.

In summary, our results confirmed that knockdown of *GhDRP* could transform a resistant accession into a susceptible one. Therefore, *GhDRP* may play an important role in the resistance of cotton to *V. dahliae*.

## Discussion

### QTLs and Candidate Genes Can Be Rapidly Mined and Screened Using the F_2:3_ Segregation Population and BSA-Seq Analysis

Upland cotton is one of the most widely cultivated cotton species in the world. It has the advantages of high yield and adaptability, but with a relatively weaker resistance to VW. Cotton VW is a soil-borne fungal disease caused by *V. dahliae* and the most serious disease in cotton production to affect both the yield and fiber quality. Numerous studies have been performed to identify the QTLs for VW resistance in cotton using different populations such as F_2_ segregation population, RIL, and backcross population with the protocols of SSR, SNP, or GWAS (Jiang et al., 2009; Zhang et al., 2014b, 2015; Shi et al., 2016; Palanga et al., 2017; Abdelraheem et al., 2019). However, these QTLs have been seldom used in breeding. Michelmore et al. (1991) for the first time used the BSA method to screen three markers closely associated with the downy mildew resistance gene DM5/8 in an isolated lettuce population. With the development of high-throughput sequencing technology, BSA-seq analysis has been applied in wheat (Yin et al., 2018), soybean (Song et al., 2017), tomato (Zhao et al., 2016), rice (Ma et al., 2018; Liang et al., 2020), groundnut (Pandey et al., 2017), pepper (Lee et al., 2020), and cotton (Chen et al., 2015; Zhu et al., 2017; Zhao et al., 2018) for the rapid mining of QTLs and screening of candidate genes. However, the method has not been used in research related to VW resistance of cotton.

In the present study, an F_2:3_ segregation population was planted in a field in Xinjiang and a greenhouse in Anyang. There was a significant correlation between RDI at seedling stage in the greenhouse and adult stage in the field, which indicated that the RDI investigated at seedling stage could represent the resistance of cotton. Extremely resistant and susceptible materials with stable performance in both environments were selected, providing a material basis for BSA-seq analysis to mine the QTLs related to cotton VW resistance. Two QTLs (qvw-D05-1 and qvw-D05-2) related to VW resistance were obtained by BSA-seq analysis, which have large mapping intervals and many candidate genes. However, it is still necessary to develop molecular markers or bioinformatics methods to further fine-map the QTLs or mine the candidate genes.

We found that SnpEff could effectively annotate variations and predict the influence of genetic variations, which may facilitate the quick anchoring of a wide range of SNP mutation sites. Therefore, we performed SnpEff analysis and transcriptome analysis to further fine-mine the candidate genes. Cingolani et al. (2012) took the SNPs in the genome of *Drosophila melanogaster strain w^1118^* as an example to elaborate and verify the principle and application of the SnpEff software. They identified 28 stop-gained SNPs and five stop-lost SNPs, which may have a great influence on gene expression. Some other researchers analyzed the genotype diversity of *Brassica napus*, potato and tomato using SnpEff as well (Kevei et al., 2015; Malmberg et al., 2018; Caruana et al., 2019). In the present study, SnpEff was used to narrow the QTL (qvw-D05-2) interval from 4.69Mb to 1.32Mb and decrease the number of candidate genes from 262 to 17. Hence, this study provides and verifies an analytical method for rapid mining of candidate genes.

### *GhDRP* Can Be Used as a Candidate Gene for VW Resistance

In this study, *GhDRP* was identified to be associated with VW resistance. Its DNA length was 7,558bp, including six exons and five introns. The total length of the CDS sequence was 3,876bp and the protein sequence contained 1,291 amino acids, including one NB-ARC and seven LRR domains. These results suggest that *GhDRP* belongs to the NBS-LRR family and its homologue in *Arabidopsis thaliana* was annotated as a disease resistance protein. NBS-LRR genes are the most important resistance gene family in plants, with the largest number of members and the most abundant types of subfamilies. Generally, it has been considered that the LRR domain plays a specific role in pathogen recognition and an important role in downstream signal transduction (Anderson et al., 1997; Parker et al., 1997; Luck et al., 2000; Dangl and Jones, 2001). The NBS-LRR genes are ubiquitous in plants: there are approximately 149 NBS-LRR genes in *Arabidopsis* (Meyers et al., 2003), 500 in rice (Monosi et al., 2004), 245 in *Solanum pimpinellifolium* (Wei et al., 2020), 333 in *Medicago truncatula* (Ameline-Torregrosa et al., 2008), 99 in *Raphanus sativus L*. (Ma et al., 2021), 400 in *Populus trichocarpa* (Kohler et al., 2008), 459 in grapevine (Yang et al., 2008), 330 in poplar (Yang et al., 2008), and 536 in upland cotton.^2^ These R genes play very important roles in plant disease resistance. For example, *GbRVd*, *GbaVd1*, *GbaVd2*, *GhPGIP1*, and *GbaNA1* have been cloned from *G. barbadense* or *G. hirsutum* and verified to be involved in the process of plant resistance to VW (Yang et al., 2016; Chen et al., 2017; Liu et al., 2017; Li et al., 2018).

In the present study, the qRT-PCR results indicated that after *V. dahliae* infection, there is a quick induction of *GhDRP* and high expression level during 36–72 h in ZZM2, while that in J11 roots was only significantly up-regulated at 72h (Figure 4B). There was the different induction pattern of *GhDRP* in resistant cultivar ZZM2 and susceptive cultivar J11. In some other studies, *GhARPL18A-6*, *GhDSC1*, and *GhCAMTA3*, which had been proved to be related to cotton disease resistance, were up-regulated especially during the early infection stages 6–24h after inoculation with *V. dahliae* in resistant cultivar cotton, but no induction was observed in susceptible cultivar (Li et al., 2019; Zhang et al., 2019a), which has the similar induction pattern with *GhDRP*. Therefore, we infer that activation of cotton immune response may depend on the duration higher expression of resistance gene instead of short-duration stress response or non-expression. Furthermore, *GhDRP* was cloned from ZZM2 and J11, and it was found that one base change in the DNA sequence led to the change of one amino acid, resulting in the production of proline in ZZM2 and glutamine in J11. Proline is a non-polar R-based amino acid with very low solubility in water and an isoelectric point of 6.30. Glutamine is an uncharged polar R-based amino acid easily soluble in water with an isoelectric point of 5.65. However, it remains unclear whether the alteration of this amino acid is related to the difference between resistant and susceptible varieties of *V. dahlia* infection, which requires further validation. Next, we will focus on developing molecular markers to validate the mutant sites in other populations, and expect to differentiate disease resistant/susceptible materials through genotyping.

The results of the VIGS experiment revealed that the plants with the silencing of *GhDRP* were more susceptible to *V. dahliae* infection than the control according to the phenotypes of plants, DI, fungal biomass, stem recovery assay, and trypan blue staining, indicating that *GhDRP* may play an important role in resistance to *V. dahliae*. However, the molecular mechanism underlying the effect requires further exploration. Furthermore, cells in TRV::00 plants showed a higher level of lignification than those in TRV::*GhDRP* plants after pathogen inoculation, suggesting that the resistance of the plants to *V. dahliae* infection may be mainly dependent on lignification, which can help to prevent fungal invasion by increasing the mechanical barrier of cell walls (Hückelhoven, 2007). It has been previously confirmed that the lignin content is positively correlated with cotton resistance to *V. dahliae* (Shi et al., 2012; Guo et al., 2016; Zhang et al., 2019b). However, the molecular mechanism and signal pathway *GhDRP* involved in remains to be elucidated. And we will conduct further functional verification of *GhDRP* by transferring the gene into cotton, and clarify its metabolic pathway and mechanism of action. In summary, *GhDRP* may be used as a candidate gene for VW resistance and has potential application in the breeding of VW resistant cotton varieties.

## Data Availability Statement

CNGB Sequence Archive (CNSA) of China National GeneBank DataBase (CNGBdb; https://db.cngb.org/) with accession number CNP0001983.

## Author Contributions

YC, QG, HW, and QC conceived and designed the experiments. YC implemented the experiments and prepared the manuscript. PZ guided the molecular experiments. WC and XS collected the field data. QG and YC analyzed the results. QG, PZ, YZ, and HW revised the manuscript. All authors contributed to the article and approved the submitted version.

## Funding

This research was supported by Natural Science Foundation of Henan Province (No. 202300410548) and Central Public-Interest Scientific Institution Basal Research Fund (No. 1610162021005).

## Conflict of Interest

The authors declare that the research was conducted in the absence of any commercial or financial relationships that could be construed as a potential conflict of interest.

## Publisher’s Note

All claims expressed in this article are solely those of the authors and do not necessarily represent those of their affiliated organizations, or those of the publisher, the editors and the reviewers. Any product that may be evaluated in this article, or claim that may be made by its manufacturer, is not guaranteed or endorsed by the publisher.

## Footnotes

^1^ https://github.com/broadinstitute/gatk/releases

^2^ https://cottonfgd.org

## Supplementary Material

Supplementary Figure 1 Cloning and differential expression of *GhDRP* in ZZM2 and J11.

Supplementary Table 1 Primer sequences used in the study.

Supplementary Table 2 RDI of individuals in resistant and susceptible pool in the field and greenhouse environments.

Supplementary Table 3 Quality evaluation of raw data and clean data.

Supplementary Table 4 Alignment assessment.
