# Supplementary figures and images for: Rapid Mining of Candidate Genes for Verticillium Wilt Resistance in Cotton Based on BSA-Seq Analysis
Source: Front Plant Sci. 2021 Oct 8;12:703011. doi: 10.3389/fpls.2021.703011 (PMC8531640; doi:10.3389/fpls.2021.703011)

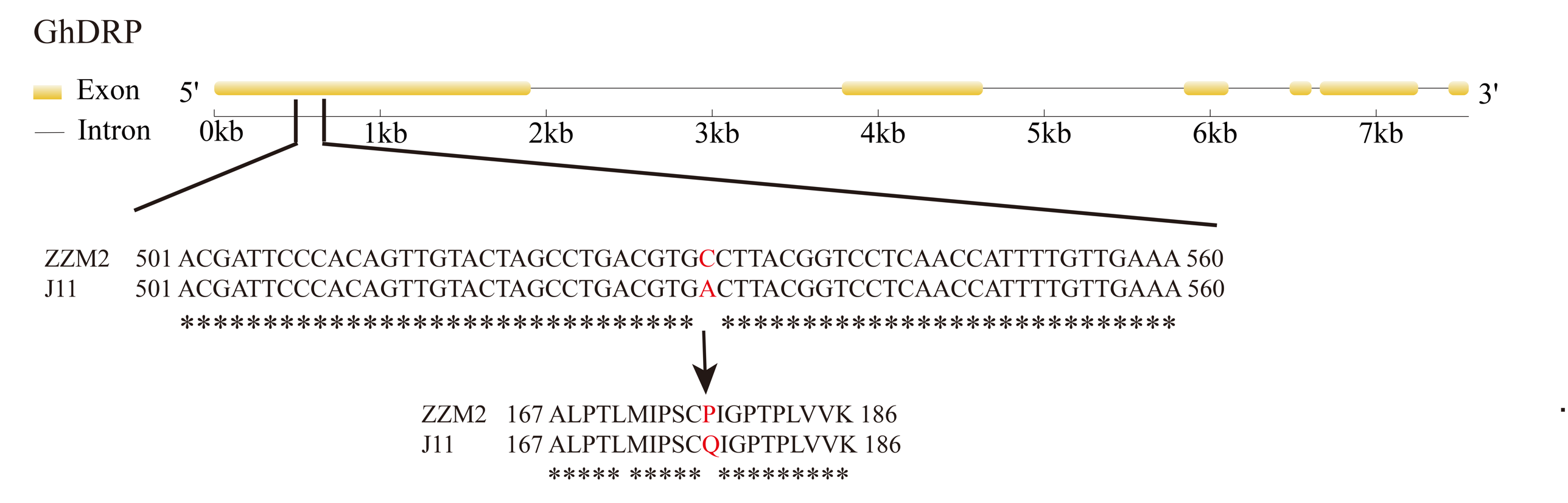

Supplement: Supplementary Figure 1 — Cloning and differential expression of GhDRP in ZZM2 and J11. [file Image_1.TIF]
