# Supplementary material for: Rapid Mining of Candidate Genes for Verticillium Wilt Resistance in Cotton Based on BSA-Seq Analysis
Source: Front Plant Sci. 2021 Oct 8;12:703011. doi: 10.3389/fpls.2021.703011 (PMC8531640; doi:10.3389/fpls.2021.703011)
Supplement: Supplementary Table 1 — Primer sequences used in the study. [file Table_1.docx]

Supplementary Table 1 Primer sequences used in the study

| Primer name | Primer sequence 5’-3’ | Notes |
| --- | --- | --- |
| Ghir_D05G037630-1F | ATGGCCGAATATGTTGCCCCTGCTG | 1-2637bp amplication of *Ghir_D05G037630* |
| Ghir_D05G037630-1R | CTACACCAGAAAGACCTAAATCCAAG | 1-2637bp amplication of *Ghir_D05G037630* |
| Ghir_D05G037630-2F | CTTGGATTTAGGTCTTTCTGGTGTAG | 2612-5193bp amplication of *Ghir_D05G037630* |
| Ghir_D05G037630-2R | CTATATTGATTTGGTGCTCTAGGGAGT | 2612-5193bp amplication of *Ghir_D05G037630* |
| Ghir_D05G037630-3F | TAGCATTTTGGTGTTAGAGCGAGTG | 5139-7558bp amplication of *Ghir_D05G037630* |
| Ghir_D05G037630-3R | TCATGTAATGCGTGGTAGTTGACTC | 5139-7558bp amplication of *Ghir_D05G037630* |
| Gh_histone3F | CGGTGGTGTGAAGAAGCCTCAT | for qRT-PCR (the house keeping gene of *Gossypium hirsutum*) |
| Gh_histone3R | AATTTCACGAACAAGCCTCTGGAA | for qRT-PCR (the house keeping gene of *Gossypium hirsutum*) |
| Ghir_RT037630F | AGAAATGAAAGCCCTCCAAG | for qRT-PCR |
| Ghir_RT037630R | TGCCAATCAACGCAAGAAT | for qRT-PCR |
| Gh_V037630-2F | ggcctcgagacgcgtgagctc  ATGTCACAACCAAAAATAATCTCAACT | for VIGS |
| Gh_V037630-2R | agaaggcctccatggggatcc  ATTGCAAGATTTTAGCAGCAGAGA | for VIGS |
| VdActin-F | CTGGTATCGTGCTTGACTCTG | for qRT-PCR |
| VdActin-R | GATTTCACGCTCGGCAGT | for qRT-PCR |

Supplementary Table 2 RDI of individuals in resistant and susceptible pool in the field and greenhouse environments

| individual | Field-S | Greenhouse-S | individual | Field-R | Greenhouse-R |
| --- | --- | --- | --- | --- | --- |
| 4 | 39.47 | 36.27 | 9 | 25.02 | 29.96 |
| 28 | 40.90 | 45.19 | 13 | 21.94 | 20.36 |
| 39 | 34.84 | 46.56 | 19 | 22.09 | 19.20 |
| 42 | 43.33 | 40.16 | 20 | 21.35 | 15.79 |
| 50 | 45.31 | 39.03 | 22 | 18.43 | 23.41 |
| 51 | 36.40 | 42.89 | 26 | 25.89 | 23.32 |
| 53 | 37.70 | 39.44 | 30 | 24.45 | 25.09 |
| 55 | 41.77 | 42.05 | 32 | 25.72 | 16.03 |
| 57 | 39.45 | 46.02 | 60 | 25.26 | 25.60 |
| 69 | 37.07 | 36.75 | 86 | 23.20 | 24.47 |
| 121 | 35.98 | 36.40 | 113 | 23.43 | 15.98 |
| 128 | 35.62 | 36.38 | 116 | 22.08 | 20.08 |
| 149 | 33.44 | 45.63 | 117 | 22.66 | 20.95 |
| 152 | 38.10 | 35.36 | 122 | 16.31 | 19.55 |
| 167 | 34.81 | 40.44 | 125 | 23.29 | 26.53 |
| 199 | 33.71 | 35.25 | 129 | 21.57 | 21.67 |
| 71 | 34.86 | 31.77 | 144 | 21.81 | 24.92 |
| 119 | 44.80 | 33.43 | 102 | 20.37 | 23.00 |

Note: Greenhouse-R stands for the RDI of 18 resistant individuals in the greenhouse. Field-R stands for the RDI of 18 resistant individuals in the field. Greenhouse-S stands for the RDI of 18 susceptible individuals in the greenhouse. Field-S stands for the RDI of 18 susceptible individuals in the field.

Supplementary Table 3 Quality evaluation of raw data and clean data

| Sample | RawBases  (bp) | CleanBases  (bp) | Q20(%) | | Q30(%) | | GC(%) | |
| --- | --- | --- | --- | --- | --- | --- | --- | --- |
|  |  |  | Raw | Clean | Raw | Clean | Raw | Clean |
| Gh_F_2_R | 77.92 | 72.36 | 96.25 | 97.48 | 90.25 | 92.19 | 35.12 | 35.21 |
| Gh_F_2_S | 78.33 | 71.67 | 95.51 | 96.97 | 88.66 | 90.93 | 34.98 | 35.08 |
| ZZM2 | 80.62 | 80.62 | 98.55 | 98.55 | 95.96 | 95.96 | 35.39 | 35.39 |
| J11 | 69.54 | 69.54 | 98.5 | 98.5 | 95.82 | 95.82 | 35.18 | 35.18 |

Supplementary Table 4 Alignment assessment

| Sample | Coverage_rate(%) | Map_reads_rate(%) | Map_bases_rate(%) | Uni_hit_reads_rate(%) | Uni_hit_bases_rate(%) | Sequencing_depth | Effective_depth |
| --- | --- | --- | --- | --- | --- | --- | --- |
| Gh_F_2_R | 94.94 | 99.55 | 99.55 | 83.86 | 83.49 | 30.82 | 30.82 |
| Gh_F_2_S | 94.97 | 99.8 | 99.8 | 84.38 | 84.22 | 30.52 | 30.52 |
| ZZM2 | 94.9 | 99.42 | 99.42 | 66.8 | 66.41 | 34.33 | 34.33 |
| J11 | 94.88 | 99.81 | 99.81 | 70.44 | 70.31 | 29.61 | 29.61 |
